# How Should the Social Service Quality Evaluation in South Korea Be Verified? Focusing on Community Care Services

**DOI:** 10.3390/healthcare8030294

**Published:** 2020-08-24

**Authors:** Kichan Yoon, Gyubeom Park, Munjae Lee

**Affiliations:** 1 Social Security Information Institute, Social Security Information Service, Seoul 04554, Korea; ykichan@ssis.or.kr (K.Y.); bum0808@ssis.or.kr (G.P.); 2 Department of Medical Device Management and Research, SAIHST, Sungkyunkwan University, Seoul 06351, Korea

**Keywords:** social service quality, social service quality evaluation system, care service, public service quality, Korea

## Abstract

The quality evaluation (QE) of social services tends to have a large variation in results depending on the object and method of service measurement. To overcome these limitations, an analysis of the internal consistency or validity of the social service QE index is necessary, but meta-research on this is insufficient. This study analyzes the internal consistency and validity of evaluation indexes based on the results of social service QE. We utilized the social services QE manual of the Social Security Information Service’s Facility Evaluation Department. The social service QE indexes implemented in 2013 and 2016 were coded and analyzed. We found that there was internal consistency between the results of the care services evaluation in 2013 and 2016. In addition, there were differences between the care services QE indexes by service type in 2013 and 2016. It is necessary to construct effective indexes by simplifying, diversifying, and differentiating social service QE indexes. In addition, control devices for external factors (region, composition of the evaluation team, etc.) must be prepared to maintain the consistency of evaluation scores, and in the long term, standardization of social service QE indexes is necessary.

## 1. Introduction

Social service quality indicates the degree to which the needs of service users are satisfied [1,2]; in quality control for social services, quality should be assessed and managed in a macroscopic and broad social context and should include the assessment of individual services [3,4,5]. In particular, for the quality control of social services, separate interventions in service quality are required to meet the users’ quality expectations. To protect the rights of service users, ensure public good, and prevent the adverse selection of suppliers, etc., quality control efforts are vital [6,7,8].

Among social services, care services could be seen as a better alternative than facility admission, in terms of the right guarantee and accessibility to services. Accordingly, developed countries, including the United States, are providing incentives to expand care services [9,10]. With respect to care services in Korea, the country mainly provides financial support for social services, such as services for postpartum women and infants, home and health (H&H) help, elderly care, etc., which could have high public responsibility as a public service model [11,12].

In Korea, since 2010, as in the US, the work of facility evaluation has been entrusted to the Social Security Information Service under the non-profit central office group. A pilot project for the QE of social services has been promoted; after the Act on the Use of Social Services and the Management of Social Service Vouchers was enacted in 2011, the QE project for social services started in 2012. As of 2018, more than six QEs of social services have been conducted by the Social Security Information Service (SSIS). In terms of care service projects, excepting regional investment projects, evaluations were performed in 2013 and 2016, and the data related to the evaluation results have been accumulated. As of 2019, QEs of social services have been implemented for care services, and work to improve the QE indexes is being executed in multilateral ways. Nevertheless, in the case of Korea, there is no certification system by meeting certain indicators, unlike the US, and it does not have any service quality standards, unlike the UK. It is staying at a level of ranking A, B, C, and D through an every 3-year social service QE. Therefore, it is difficult to guarantee the reliability and validity on the QE because the indexes are varying for each evaluation period [13,14,15]. 

In the QE of social services, there is a tendency for the deviation in the results to increase depending on the measurement targets and methods if the service contents are professional or the service users are relatively vulnerable to service information [16,17]. Moreover, there are aspects in which service quality differs depending on the characteristics of the provided social service types or organizations, and it is difficult to reflect this in evaluations [17,18]. Although the social service QE system has recently entered a period of settlement, there is a limit to the QE of social services, and there is demand for improvement. There has not been an in-depth analysis of the differences between the 2013 and 2016 social service QE results, which were conducted by the Social Security Information Service, or by service type and institution type. In addition, research on internal consistency and validity in the QE indexes of social services is lacking; in particular, the meta-studies lack QE of social services, especially those centered on care services [19]. Therefore, social demands for improvement are emerging so that the current social service QE can be applied practically.

Korean social services have marketability and are public at the same time. Although the private sector holds most social service providers, most of the resources required in the process of providing social services are public services funded by the government and local governments. As social services based on public sector finances occupy an absolute amount, the importance of the public sector and QE for social services is emphasized at the same time. However, the QE of social services is being operated as a private for-profit model transferred to private institutions. Like the US and the UK, Korea transferred the QE of social services to private institutions, but the government’s regulatory level is not as high as theirs. Also, although public institutions oversee social services, like in Sweden, a strong national QE system is insufficient. Therefore, it is judged that systematic indexes that can perform practical evaluations are necessary for the QE of social services.

Thus, in this study, we analyzed the internal consistency and validity of QE indexes based on the results of the QE of social services that were conducted twice, focusing on care services. Through this, we intended to highlight the problems with the QE indexes of social services, including care services, and then propose improvement directions for these indexes in the future. The results of this study serve as a basis for the standardization of social service QE indexes and can help establish a strong, national-level guideline to ensure the quality of social services. The design of such evaluation criteria will derive evaluation indexes suitable for global standards and can improve the quality of social services by resolving user complaints and reinforcing options.

## 2. Materials and Methods

### 2.1. Definition of the Variables

Among the QEs of social services implemented and centered on the SSIS in 2012, service QEs were conducted in 2013 and 2016 on care services, including those for postpartum women and infants, H&H help, elderly care, etc. 

The quality of care service was evaluated based on the evaluation indexes that measure its quality. Also, based on the QE results, the evaluation of the care service institution and the improvement plan for institutional operation are derived. Considering the results of the QE of social services conducted in 2013 and 2016, it was found that the evaluation results of institutions, which have received a field evaluation in 2013 (1st period), were superior to those of the non-evaluated institutions, demonstrating a learning effect from evaluation. Since a majority of projects have been increased or continuously kept the same social service QE grades, it is necessary to examine the internal consistency of the evaluation results. Also, it is necessary to analyze the difference in evaluation index scoring as projects whose rating grades have increased vary by service type.

Meanwhile, the comparative analysis of social service QE indexes for care services, conducted in 2013 and 2016 showed that a total of 14 indexes, including six indexes in institutional operations, five in human management, and three in service areas, were consistent. First, in terms of institutional operations for operating systems, the operational regulations and operating plans were derived as common indexes; for information management, information protection and information security were used as common indexes; in accounting management, accounting management and settlement disclosure were common indexes; there was no common index in project evaluation and publicity. Second, in terms of the manpower management sector within human resource management, the recruiting process, labor contracts, and standard compliance were common indexes; in the educational system, education time was applied in common. Common indexes did not exist in business control, educational content, and right guarantee. Third, in the case of service environment among service areas, attire management was a common index, and in terms of tenure rate area, the tenure rate belonged to a common index also. In establishment of plans, the counseling plan and record management appeared as a common index; user satisfaction was commonly used in implementation and monitoring. In the sector of service linkage and termination, contract termination and document filing were applied as common indexes. Fourth, in the field evaluation area, the field evaluation itself was commonly used in both 2013 and 2016. In view of this, we judged that it is necessary to analyze by matching the evaluation weights in the years of 2013 and 2016 based on common indexes.

**Figure 1:**
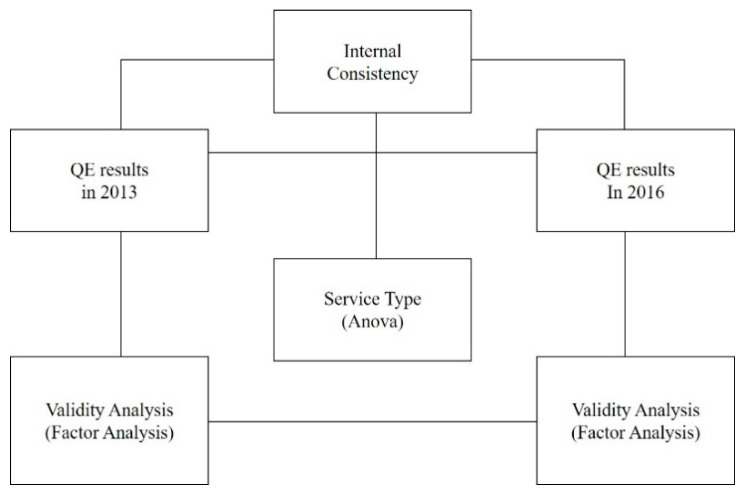
Framework of the research. QE: quality evaluation.

In this study, we derived the improvement directions and priority order for QE indexes of social services by analyzing the internal consistency and validity of the social service QE indexes of care services, which have been conducted twice. The analysis framework is as follows. A paired *t*-test was conducted to analyze the internal consistency between the QE results of 2013 and 2016. To analyze the validity of the QE indexes of care services from 2013 and 2016, a factor analysis was utilized. To analyze the difference between the QE results by profit type and service type in 2013 and 2016, we utilized an analysis of variance (Figure 1).

The hypotheses for this study are as follows:

**Hypothesis** **1a.** *There will be internal consistency between the QE results of care services in 2013 and those in 2016.*

**Hypothesis** **1b.** *There will be internal consistency between the QE indexes of care services by service type in 2013 and those in 2016.*

**Hypothesis** **2.** *There will be individual validity in the QE indexes of care services in 2013 and 2016.*

### 2.2. Method

Internal consistency analysis is a method to evaluate reliability, which divides one measurement tool into two—each having the same number of questions—and then evaluates the correlation between the two overall scores [20]. The methods of evaluating internal consistency include the confidence coefficient (Cronbach’s α) and Cohen’s Kappa coefficient, etc. The confidence coefficient has a value of 0 to 1 in the reliability evaluation tool of the question; values of 0.7 or higher are considered to have high reliability. Cohen’s Kappa coefficient is a method used to measure the reliability of two evaluators; a value of 0.6 or higher can be considered as having consistency [21]. In this study, as a result of measuring Cronbach’s α and the Kappa coefficients in advance, significant results were not obtained. Accordingly, a test–retest method of reliability evaluating methods was used. In other words, internal consistency was evaluated by comparing the consistency of the results at each time with the average of the internal consistency rating for each care service type, targeting 423 institutions that have the same indexes and that commonly became the evaluation subjects both in 2013 and 2016 [20,22]. Validity evaluation is a concept that indicates whether a particular index sufficiently reflects the actual meaning of the considered concept [23]. The methods to measure validity include Content Validity, Construct Validity, Criterion Validity, etc. [24]; in this study, factor analysis was utilized to verify construct validity. Factor analysis is a method of classifying multiple interrelated variables into a more limited number of common factors. Factor analysis includes Exploratory Factor Analysis and Confirmatory Factor Analysis; this study analyzed what common factors the common evaluation indexes in 2013 and 2016 are grouped by [25].

Therefore, in this study, the following research methods were used to analyze the internal consistency and validity of the QEs of social services based on care services. First, we attempted to use the Kappa coefficient to analyze the internal consistency of the QE of social services in 2013 and 2016, but no significant value was derived; therefore, we conducted a paired *t*-test on the concerted QE indexes for 2013 and 2016. As a result of the analysis, it was judged that if the score of the evaluation indicator in 2016 was improved compared to that in 2013, there was an internal consistency. This is because it was considered that the learning effect on the previous evaluation indexes appeared.

Second, to analyze the internal consistency by service type (postpartum women and infants, H&H help, and elderly care), an ANOVA was performed. Scheffé’s method was adopted for the post-hoc validation of the ANOVA. As a result of the analysis, if there was a difference in averages for each service type, it was considered that the internal consistency was low. It is because the reliability of the evaluation indicator lowers if the evaluation score varies by service type. 

Third, to analyze the validity of the QE index of care services, we verified the degree of validity of the factors, such as institutional operation, human management, service area, and field evaluation, using factor analysis.

### 2.3. Data Collection

In the data collection stage, we utilized the manual of QE of social services by the Facility Evaluation Department of the SSIS, and the secondary data were quantified based on the analysis results [26,27]. To this end, in connection with the information held by the SSIS, we newly coded scores for indexes commonly used for evaluations in both 2013 and 2016. To analyze the internal consistency and validity between the QE indexes in 2013 and 2016 for care services among the social services, the following steps were conducted, centered on common indexes:

For institutional operation, 1 point was commonly applied to operational regulations, operation plan, information protection, information security, accounting management, and settlement disclosure.

For human management, the recruitment process and labor contracts were unified into 1 point; the standard compliance and education time were assigned 1 and 2 points, respectively, in accordance with the mark distribution criteria of 2013.

In terms of service area, 1 point, 3 points, 1 point, 1 point, 1 point, and 1 point were assigned to attire management, tenure rate, counseling plan, record management, community, and contract termination, respectively, according to the criteria of 2013; 1 point, the same distribution criterion, was applied to contract termination. In the case of satisfaction, as the difference in the allotment of points was excessively large (1 point for 2013 and 25 for 2016), only 1 point was commonly applied to it.

Fourth, in the case of field evaluations, 6 points were allotted based on the point distribution criteria in 2013. The specific common indexes for QEs of social services are listed in Table A1.

## 3. Results

### 3.1. Status of Evaluation Target Institutions

In order to evaluate the quality of social services, the evaluation was performed based on the data inputted on the information system, and these data were collected through the facility evaluation information system of the SSIS. Therefore, the QE on care services was also executed by the SSIS. The status of specific providers is as follows. In 2016, the target institutions for the social service QE were total 705, in the order of 409 elderly care institutions, 202 postpartum women and infants care institutions, and 94 home and health (H&H) help ones. The number of institutions providing elderly care and postpartum women and infants services in Gyeonggi-do was 64 and 53, respectively, which accounted for the most of these institutions; Jeollabuk-do had the largest number of institutions providing H&H services, with 14 institutions. Since it was based on the entire number service providers in 2013, there were more providers than those in 2016, but its national distribution shows a similar tendency. Since this study measures the internal consistency of the indexes that measure the quality of social services, the number of institutions was not expected to have a significant effect. Elderly care and H&H services have high proportions in metropoles and rural areas, and it is judged that both the number of absolute populations and the proportion of the elderly are considered to have an impact on them. In the case of postpartum women and infants, the proportion in the metropolitan area, where 40% of the total population is concentrated, appeared high (Table 1).

### 3.2. Internal Consistency of the QEs of Social Services

#### 3.2.1. Internal Consistency between 2013 and 2016

To verify the internal consistency of the common indexes of QEs of social services in both 2013 and 2016, a paired *t*-test was performed. An analysis was conducted on whether the quality of social service was consistently measured by verifying the internal consistency of the indexes that evaluate the quality of service of social service providers. The result showed that if there was a statistically significant difference between the QE results of 2013 and 2016, it would be difficult to judge the internal consistency of the indexes (Table 2).

First, the analysis showed that the number of users and sales was significantly different in performance. The number of users and sales increased in 2016, compared to 2013. Most social services in Korea are provided by private institutions, and the sales of the institutions are related to human resource management and institutional operation. Therefore, an increase in sales seems to have improved the quality of social services and ultimately increased the number of users. Second, in terms of institutional operation, there were significant differences in the rest of the evaluation indexes, except for operation plans, and the evaluation score was higher in 2016. It could be seen that a learning effect existed in evaluation indexes under the 3-year cycle evaluation process. Third, in the case of human resource management, there were significant differences in recruitment process, period compliance, and education time, but not in labor contracts. However, in terms of period compliance and education time, the evaluation score appeared lower in 2016 than in 2013, showing low internal consistency in these evaluation indexes. Fourth, in the case of service sectors, there were improvements in the 2016 evaluation scores in attire management, tenure rate, record management, and contract termination, but the scores of satisfaction and community linkage were lower than those in 2013. Fifth, it could be seen that even in the case of field evaluations, the QE indexes in 2016 had improved over those in 2013. 

In conclusion, the comparison of the internal consistency of QE indexes in both 2013 and 2016 via a paired *t*-test showed no change in operation planning in institutional operation, labor contracts in human resource management, and counseling planning and document filing. In contrast, most of the evaluation indexes showed a statistically significant increase due to learning effects, etc., but standard compliance, education time, etc., in human resource management and satisfaction and community linkage in the service area showed lower evaluation scores in 2016 compared with 2013, which in general contributed to lowering the internal consistency of those QE indexes.

#### 3.2.2. Internal Consistency by Service Type

To compare internal consistency in the evaluation scores for each type of service (e.g., postpartum women and infants, H&H help, and elderly care services), a one-way batch analysis was conducted. We tried to pursue a diversity of evaluation indicators by confirming what difference the social service evaluation score represents for each service type (measured by the QE index) and confirming what factors impact the service evaluation score.

First, the mean difference by service type was analyzed based on the QE results of social services in 2013. The results showed that services for postpartum women and infants had a higher number of users, and in the case of sales, services for postpartum women and infants and elderly care were higher than H&H help.

Second, in operational planning, information protection, information security, accounting management, accounting disclosure, etc., in the institutional operations area, the services of H&H help and elderly care had higher evaluation scores than services for postpartum women and infants. Third, in recruitment process, labor contract, standard compliance, etc., in the human resource management area, the evaluation scores of H&H help and elderly care services appeared higher than those of services for postpartum women and infants. 

Third, in attire management, tenure rate, record management, community, contract termination, education time, etc., in the service area, the evaluation scores of H&H help and elderly care services were statistically higher than those of services for postpartum women and infants. Fourth, in field evaluation, the evaluation scores of H&H help and elderly care services also appeared higher than those of services for postpartum women and infants. The fact that an inconsistency in the evaluation scores was visible among three separate service areas means that it should be considered to apply differently to evaluation indexes. In particular, in the case of postpartum women and infants, it is necessary to develop evaluation indexes suited to the service characteristics through adjustment of the evaluation indexes (Table 3).

Next, we compared and analyzed internal consistency of each service area for postpartum women and infants, H&H help, and elderly care in the QE of social services in 2016. The analysis results were as follows. First, the number of service users was higher in services for postpartum women and infants. In terms of sales, the evaluation scores of services for postpartum women and infants and elderly care appeared higher than those of H&H help services. Second, in the case of institutional operations, in the evaluation indexes of operational regulations, information security, settlement disclosure, etc., the evaluation scores of the services of H&H help and elderly care were higher than those of services for postpartum women and infants. Third, in terms of the human resource management area, in labor contracts, education time, etc., the evaluation scores of services for postpartum women and infants appeared lower than those of H&H help and elderly care. Fourth, in terms of the service area, in satisfaction, community linkage, contract termination, etc., the evaluation scores of H&H help and elderly care services were higher than those of services for postpartum women and infants. Fifth, even in the field evaluation, the evaluation scores of H&H help and elderly care services appeared higher than those of services for postpartum women and infants. In conclusion, they were of low internal consistency by service types. Thus, also in the case of social service QEs by service type in 2016, there is a need to specialize in the evaluation indexes of services for postpartum women and infants, and it is vital to develop differentiated evaluation indexes by service type (Table 3).

### 3.3. Validity of QE Indexes of Social services 

A factor analysis was conducted to verify the index validity of the QE of social services, implemented in both 2013 and 2016. The method of principal component analysis was selected, and for the rotation method the varimax method was used. We verified the validity of the evaluation indexes in three areas, institutional operation, human resource management, and service area, with the exception of field evaluation, among the QE indexes of social services.

A factor analysis was conducted on the QE indexes of 2013; the Kaiser-Meyer-Olkin (KMO) value was higher than 0.5, indicating that the evaluation indexes were appropriate for a factor analysis. Since the KMO value was 0.807 and the *p*-value of Bartlett’s test was 0.001, using a factor analysis was considered proper. In Factor 1, operational plans, operational regulations, information protection, and information security, which were related to institutional operation, were unified into a single factor that included evaluation indexes such as recruitment processes, etc. in human resource management and community, attire management, etc. in the service area. In Factor 2, standard compliance, education time, labor contracts, etc. in human resource management were integrated into a single factor that contained accounting management, settlement disclosure, etc. in institutional operation. Factor 3 consisted of document filing, contract termination, satisfaction, counselling plan, and record management in the service area as a single factor. Thus, we judged that the QE of social services in 2013 was low in terms of the validity of the evaluation indexes in the areas of institutional operations and human resources (Table 4).

Meanwhile, the reliability coefficient (Cronbach’ α) k, measuring the reliability of the evaluation indexes, was measured. Cronbach’ α refers to the ‘High Stakes Testing’ if it is 0.9 or higher, and the ‘Low Stakes Testing’ if it is 0.7 or higher. It could be seen as acceptable only when it becomes at least 0.6 or higher. Factor 1 was 0.694, Factor 2 was 0.264, and Factor 3 (service area) was 0.478; even Factor 3 (service area), which appeared to be relatively valid, did not have any reliability.

A factor analysis was conducted to verify the validity of institutional operation, human resource management, and services, which belong to the QE index of social services in 2016. As the KMO value was 0.813 and the *p*-value of Bartlett’s test was 0.001, using a factor analysis was considered proper. In Factor 1, operational regulations, operational plans, information security, and information protection in the institutional operations area were unified into a single factor. It included education time in the area of human resource management and attire management, and contract termination, document filing, community, etc., in the service area. Factor 2 included settlement disclosure in the institutional operation area; labor contracts, recruitment process, etc., in the area of human resource management; and tenure rate and satisfaction in the service area. Factor 3 comprised of record management and counseling contracts in the service area and accounting management, etc. in the institutional operations area as a single factor. In conclusion, the QE indexes of social services in 2016 had a lower construct validity compared with 2013; thus, it is difficult to distinguish the evaluations for the three areas (Table 4).

In the meantime, as a result of measuring the reliability coefficient (Cronbach’ α) k to measure the reliability of the evaluation indexes, it was found that Factor 1 was 0.643, Factor 2 was 0.311, and Factor 3 was 0.280. In other words, the social service QE indexes in 2016 did not show both validity and reliability.

## 4. Conclusions

In this study, to analyze the internal consistency and validity of the social service QE system, we utilized evaluations of care services performed in 2013 and 2016. For the research data, we used the QE results of services for postpartum women and infants, H&H help, and elderly care, which were executed by the SSIS in 2013 and 2016, and we selected and utilized the indexes that were commonly applied in both years. In terms of the research method, to verify the internal consistency on the social service quality system, a paired *t*-test and ANOVA were implemented; for the validity analysis, a factor analysis was used. 

First, as a result of the analysis, Hypothesis 1a was accepted. In other words, after comparing the internal consistency of the QE indexes in 2013 and 2016 through the paired *t*-test, it was found that most of the evaluation indexes showed a significant increase in their evaluation scores due to learning effects or the like. However, standard compliance and education time in human resource management, and satisfaction and community linkage in the service area, had lower evaluation scores. It was found that the social service evaluation score conducted in 2016 increased compared to that of 2013. In particular, the scores for the variables of performance and institutional operation increased. Service institutions that received social service QE in 2013 improved service quality through supplementation. In other words, it is judged that the evaluation score has increased due to the learning effect of social service institutions.

Second, Hypothesis 1b was rejected. The QE score by service type for services for postpartum women and infants appeared lower than those of H&H help and elderly care services. These results were derived because regional differences were not reflected when evaluating user satisfaction and there were differences in the users’ characteristics [28].

Third, Hypothesis 2 was rejected. In the QE index for 2013, only the service area was found to be valid by the factor analysis and it was also found that the other areas of institutional operation and human resource management were not valid. The evaluation indexes in 2016 were found to be invalid in all of the institutional operations, human resource management, and service areas. Through this, we judged that, although the characteristics of the project users differed by service type with regard to the composition of the indexes for the QE of social services in 2013 and 2016, the validity of the evaluations was reduced by using the same evaluation indexes.

Based on the results of the internal consistency and validity analysis on the above social service QE system, the priorities for the improvement direction of the social service QE system centered on care services are as follows.

First, the QE indexes of social services should be simplified so that they provide a valid evaluation of the actual service, not just a nominal evaluation for evaluation’s sake. Unnecessary evaluation indexes should be removed and the composition of effective indexes should be discussed [29]. The Facility Evaluation Department of the SSIS abolished the settlement disclosure item, integrated the accounting management item, and repealed the document filing index in the scheme research to improve the social service QE system in 2019. In addition, it was improved so that it now measures the satisfaction of both consumers and suppliers by adding provided manpower satisfaction to user satisfaction [30].

Second, the QE indexes by service type should be diversified and differentiated. This study also found that the evaluation scores for services for postpartum women and infants were remarkably lower than those of H&H help and elderly care services. Accordingly, the Facility Evaluation Department of the SSIS intends to realize diversification of the evaluation indexes through the improvement of the indexes, by adding a visiting counseling management index to the H&H help and elderly care services and by adding a purchasing conversion rate index to the services for postpartum women and infants [22,31].

Third, it is necessary to compose a pool of QE indexes for social services and to introduce a modular approach to sorting them into essential indexes that contributed to the improvement of social service quality, and optional indexes, then to exclude the indexes of total indexes that contributed to the service quality improvement, to some extent, and add new sub-indexes to it [32].

Fourth, a control system should be in place for external factors such as regional characteristics, evaluation team composition, etc., which work as constraints on securing fairness in social service QE. In other words, the differences in the characteristics of service users in large cities and those in rural areas should be reflected in the evaluation index; sufficient training is required to maintain the consistency of the evaluation scores according to the composition of the evaluation team.

Fifth, standardization of QE indexes for social services should be attempted for strategic quality control in the long term. That is, it is necessary to establish a standardization basis for the evaluation indexes based on internal consistency and validity and to restructure the evaluation indexes to meet global standards [33].

This study attempted to provide a direction for improvement of the social service QE indexes in the future based on the social service QE, which has been conducted triennially since 2013. However, in 2019, QE was conducted by reducing detailed items at each level for the quantitative easing of social services. Due to this, there was a limit to finding common indexes using the evaluation indexes of 2013 and 2016. In future studies, it is expected that verification at an empirical level should be conducted through a comparative analysis of the 3-year social service QE indexes of 2013, 2016, and 2019. However, this study suggested directions for improvement to effectively conduct QE for social services. In particular, a direction for improving the QE system through analysis of the internal consistency and validity of the evaluation indicators can be used as basic data for the construction of a 4th social service QE model in the future. In conclusion, the framework offered here can serve as the basis for system development and operational direction for social service QE operationalization at the practical level.

## Figures and Tables

**Table 1:** Distribution status of target providing institutions of social service evaluation (unit: piece (%)).

	Number of Providing Institutions		Elderly Care		H&H Help		Postpartum Women and Infants	
Year	2016	2013	2016	2013	2016	2013	2016	2013
Seoul	57	267	21	156	6	79	30	32
Busan	46	126	29	86	8	33	9	7
Daegu	33	76	17	54	7	15	9	7
Incheon	30	68	12	43	3	14	15	11
Gwangju	32	91	22	66	7	20	3	5
Daejeon	21	50	12	37	3	8	6	5
Ulsan	15	28	6	18	4	6	5	4
Sejong	2	8	1	4	-	3	1	1
Gyeonggi	123	294	53	153	6	63	64	78
Gangwon	43	93	29	51	5	25	9	17
Chungbuk	27	61	17	37	3	15	7	9
Chungnam	36	113	25	67	3	32	8	14
Jeonbuk	59	127	40	97	14	24	5	5
Jeonnam	62	169	48	123	9	36	5	10
Gyeongbuk	57	192	41	137	8	42	8	13
Gyeongnam	55	173	33	122	6	33	16	18
Jeju	7	44	3	28	2	11	2	5
Total (unit)	705	1980	409	1279	94	450	202	242
Ratio (%)	100	100	58.0	64.6	13.3	22.7	28.7	12.2

Source: Social Service QE Result Report for each year. The number of providers in 2013 is based on the total number of providers; the number of providers in 2016 is based on evaluation results. H&H: home and health.

**Table 2:** Common indexes for QE of social services in 2013 and 2016.

Factor	Variable	*N*	2013	2016	*t*-value
Performance	Number of users	423	99.67 (128.09)	126.87 (156.71)	−5.167 *
	Sales	423	8.8141 × 10^7^ (6.30763 × 10^7^)	18.9390 × 10^8^ (11.27230 × 10^8^)	−26.423 *
Institutional operation	Operational regulations	423	0.85 (0.35)	0.93 (0.21)	−4.066 *
	Operating plan	423	0.91 (0.28)	0.94 (0.23)	−1.636
	Information protection	423	0.79 (0.41)	0.91 (0.25)	−5.440 *
	Information security	423	0.95 (0.22)	0.98 (0.11)	−2.767 *
	Accounting management	423	0.90 (0.30)	0.97 (0.15)	−4.377 *
	Settlement disclosure	423	0.82 (0.38)	0.91 (0.28)	−4.817 *
Human resource management	Recruiting process	423	0.82 (0.39)	0.88 (0.32)	−2.972 *
	Labor contracts	423	0.95 (0.23)	0.92 (0.23)	1.490
	Standard compliance	423	0.99 (0.12)	0.97 (0.16)	2.117 *
	Education time	423	1.89 (0.36)	1.80 (0.48)	3.217 *
Service area	Attire management	423	0.83 (0.37)	0.92 (0.20)	−4.659 *
	Tenure rate	423	2.19 (0.78)	2.72 (0.59)	−12.528 *
	Counseling plan	423	0.87 (0.33)	0.89 (0.23)	−0.930
	Record management	423	0.91 (0.29)	0.96 (0.16)	−3.342 *
	Satisfaction	423	0.99 (0.12)	0.92 (0.05)	10.553 *
	Community	423	0.91 (0.28)	0.81 (0.31)	5.723 *
	Contract termination	423	0.86 (0.34)	0.91 (0.29)	−2.404 *
	Document filing	423	0.96 (0.19)	0.97 (0.13)	−0.834
Field evaluation team	Field evaluation	423	4.29 (1.51)	4.84 (1.26)	−7.190 *

* *p* < 0.05. ( ): Standard Deviation. Normality Test: Here, since the samples of the paired *t*-test are the same group and the number of samples is more than 30 units, it meets normality.

**Table 3:** Difference analysis by service type in 2013 and 2016.

Classification			Division Type (2013)				Classification		Division Type (2016)			
			Postpartum Women and Infants (*n* = 84)	H&H Help (*n* = 70)	Elderly Care (*n* = 268)	F (*p*)			Postpartum Women and Infants (*n* = 84)	H&H Help (*n* = 70)	Elderly Care (*n* = 268)	F (*p*)
Performance	Number of users	Mean (SD)	219.83 ^b^ (244.48)	57.27 ^a^ (22.38)	73.08 ^a^ (37.93)	59.512 (0.001)	Number of users	Mean (SD)	338 ^b^ (249.21)	67.31 ^a^ (31.68)	76.16 ^a^ (40.36)	173.789 (0.001)
		Scheffé	a < b					Scheffé	a < b			
	Sales	Mean (SD)	91165462.90 ^b^ (1.00)	51820757.28 ^a^ (21510073.73)	96680047.27 ^b^ (50795435.53)	15.106 (0.001)	Sales	Mean (SD)	1.95 ^b^ (1.48)	1.45 ^a^ (67466663.87)	1.99 ^b^ (1.07)	6.721 (0.001)
		Scheffé	a < b					Scheffé	a < b			
	Operating plan	Mean (SD)	0.82 ^a^ (0.39)	0.94 ^b^ (0.23)	0.94 ^b^ (0.24)	6.019 (0.03)						
		Scheffé	a < b									
Institutional operation	Information protection	Mean (SD)	0.54 ^a^ (0.50)	0.84 ^b^ (0.37)	0.85 ^b^ (0.36)	21.697 (0.001)						
		Scheffé	a < b									
							Operational regulations	Mean (SD)	0.86 ^a^ (0.27)	0.99 ^b^ (0.08)	0.94 ^b^ (0.21)	7.324 (0.001)
								Scheffé	a < b			
	Information security	Mean (SD)	0.88 ^a^ (0.33)	0.96 ^a^ (0.20)	0.97 ^b^ (0.18)	4.904 (0.008)	Information security	Mean (SD)	0.94 ^a^ (0.18)	1.00 ^b^ (0.01)	0.99 ^b^ (0.09)	8.086 (0.001)
		Scheffé	a < b					Scheffé	a < b			
	Accounting management	Mean (SD)	0.74 ^a^ (0.44)	0.90 ^b^ (0.30)	0.95 ^b^ (0.22)	16.534 (0.001)						
		Scheffé	a < b									
	Settlement disclosure	Mean (SD)	0.51 ^a^ (0.50)	0.90 ^b^ (0.30)	0.90 ^b^ (0.31)	39.982 (0.001)	Settlement disclosure	Mean (SD)	0.74 ^a^ (0.44)	0.99 ^b^ (0.12)	0.95 ^b^ (0.22)	22.507 (0.001)
		Scheffé	a < b					Scheffé	a < b			
Human resource management	Recruiting process	Mean (SD)	0.58 ^a^ (0.50)	0.91 ^b^ (0.28)	0.86 ^b^ (0.35)	21.082 (0.001)						
		Scheffé	a < b									
	Labor contracts	Mean (SD)	0.82 ^a^ (0.39)	0.99 ^b^ (0.12)	0.97 ^b^ (0.16)	16.951 (0.001)	Labor contracts	Mean (SD)	1.61 ^a^ (0.73)	1.96 ^b^ (0.27)	1.89 ^b^ (0.38)	15.079 (0.001)
		Scheffé	a < b					Scheffé	a < b			
	Standard compliance	Mean (SD)	0.94 ^a^ (0.24)	1.00 ^b^ (0.01)	1.00 ^b^ (0.06)	7.968 (0.001)						
		Scheffé	a < b									
	Education time	Mean (SD)	1.68 ^a^ (0.58)	2.00 ^b^ (0.01)	1.93 ^b^ (0.28)	21.258 (0.001)	Education time	Mean (SD)	1.56 ^a^ (0.68)	1.90 ^b^ (0.30)	1.86 ^b^ (0.41)	14.796 (0.001)
		Scheffé	a < b					Scheffé	a < b			
Service area	Attire management	Mean (SD)	0.63 ^a^ (0.49)	0.89 ^b^ (0.32)	0.88 ^b^ (0.32)	16.868 (0.001)						
		Scheffé	a < b									
	Tenure rate	Mean (SD)	4.96a (2.79)	7.27 ^b^ (2.21)	6.90b (1.99)	28.804 (0.001)	Tenure rate	Mean (SD)	2.55 ^a^ (0.77)	2.80 ^b^ (0.53)	2.76 ^b^ (0.53)	5.010 (0.007)
		Scheffé	a < b					Scheffé	a < b			
	Record management	Mean (SD)	0.68 ^a^ (0.47)	0.99 ^b^ (0.12)	0.96 ^b^ (0.20)	39.014 (0.001)						
		Scheffé	a < b									
	Community	Mean (SD)	0.76 ^a^ (0.43)	0.94 ^b^ (0.23)	0.95 ^b^ (0.21)	15.942 (0.001)	Community	Mean (SD)	2.04 ^a^ (1.08)	2.61 ^b^ (0.80)	2.50 ^b^ (0.90)	9.763 (0.001)
		Scheffé	a < b					Scheffé	a < b			
	Contract termination	Mean (SD)	0.58 ^a^ (0.50)	0.94 ^a^ (0.23)	0.93 ^b^ (0.26)	41.157 (0.001)	Contract termination	Mean (SD)	0.83 ^a^ (0.37)	0.91 ^b^ (0.28)	0.93 ^b^ (0.25)	3.949 (0.02)
		Scheffé	a < b					Scheffé	a < b			
							Satisfaction	Mean (SD)	21.79 ^a^ (1.22)	22.81 ^b^ (1.42)	23.41 ^c^ (0.90)	74.516 (0.001)
								Scheffé	a < b < c			
Field evaluation		Mean (SD)	2.85 ^a^ (1.72)	4.70 ^b^ (1.15)	4.64 ^b^ (1.23)	61.303 (0.001)	Field evaluation	Mean (SD)	4.19 ^a^ (1.51)	5.15 ^b^ (1.04)	4.96 ^b^ (1.16)	15.521 (0.001)
		Scheffé	a < b					Scheffé	a < b			

There is a mean difference between the group in a and the group in b. ( ): standard deviation. ANOVA Normality Test: The number of samples for each service type is 30 or more, which meets the normality assumption.

**Table 4:** Common indexes for QE of social services (2013 and 2016).

Factor	Variable	Item		Community	Component (2013)			Item		Community	Component (2016)		
					1	2	3				1	2	3
Institutional operation	Operating plan	-	Operating plan	0.520	0.719			-	Operating plan	0.265	0.471		
	Operational regulations		Operational regulations	0.369	0.599				Operational regulations	0.560	0.743		
	Information protection		Information protection	0.409	0.562				Information protection	0.386	0.616		
	Information security		Information security	0.253	0.352				Information security	0.455	0.645		
	Accounting management		Accounting management	0.332		0.555			Accounting management	0.330			0.534
	Settlement disclosure		Settlement disclosure	0.381		0.494			Settlement disclosure	0.455		0.549	
Human resource management	Standard compliance	-	Standard compliance	0.417		0.627		-					
	Education time		Education time	0.423		0.584			Education time	0.247	0.400		
	Labor contracts		Labor contracts	0.317		0.491			Labor contracts	0.401		0.613	
	Recruiting process		Recruiting process	0.346	0.427				Recruiting process	0.308		0.391	
Service area	Document filing	Service area	Document filing	0.396			0.619	-	Document filing	0.218	0.398		
	Contract termination		Contract termination	0.513			0.561		Contract termination	0.286	0.497		
	Satisfaction		Satisfaction	0.287			0.521		Satisfaction	0.494		0.590	
	Counseling plan		Counseling plan	0.272			0.413		Counseling plan	0.422			0.462
	Record management		Record management	0.364			0.380		Record management	0.453			0.673
	Attire management		Attire management	0.391	0.565				Attire management	0.523	0.605		
	Community		Community	0.451	0.627				Community	0.332	0.375		
	Tenure rate		Tenure rate	0.365		0.598			Tenure rate	0.327		0.459	
Cronbach’α					0.694	0.264	0.478	Cronbach’α			0.643	0.311	0.280
Kaiser–Meyer–Olkin Measure					0.807			Kaiser–Meyer–Olkin Measure			0.813		
(Bartlett’s Test of Sphericity)					Approximate x^2^	1294.33		(Bartlett’s Test of Sphericity)			Approximate x^2^	1155.67	
					Freedom	153					Freedom	136	
					*p*	0.001 *					*p*	0.001 *	

* *p* < 0.05.

## Appendix A

**Table A1:** Common indexes for the quality evaluation (QE) of social services in 2013 and 2016.

Evaluation Area	Evaluation Index	2013		Evaluation Index	2016		Final Index	Point
		Detailed Index	Point		Detailed Index	Point		
Institutional Operations	Operating System	Arranging operational regulations Project operation plan	1 1	-	Institutional operating regulations Project operation plan	1 1	Operating Regulations Operation Plan	1 1
	Information Management	Privacy guidelines and education Security maintenance of personal information files	1 1	-	Personal information protection management Personal information security management	1 1	Information protection Information security	1 1
	Accounting Management	Income and expenditure entry by service Once-a-year settlement statement disclosure	1 1	-	Accounting management by service Settlement statement disclosure	1 1	Accounting management Settlement disclosure	1 1
Human resource management	Manpower management	Official recruiting process Salary provision under labor contracts Meeting required qualifications standards	1 1 1	Maintenance of recruitment	Fairness of recruitment Compliance with labor contracts Compliance with registration criteria	1 1 1	Recruitment Process Labor contract Standard Compliance	1 1 1
	Education System	In-house training for offered manpower External training for offered manpower	2	-	Yearly education time for offered manpower	2	Education time	2
Service Area	Service environment	Attire management for offered manpower	1	-	Attire management for offered manpower	1	Attire management	1
	Tenure rate	Calculation of tenure rate for offered manpower	3	-	Tenure rate for offered manpower (divisions of manpower)	3	Tenure rate	3
	Plan establishment	Service provision plan for each user Description of service provision schedule	1 1	Plan establishment Contract conclusion	Initial counseling and service provision plan Record management for service provision	1 1	Counseling plan Record management	1 1
	Execution and monitoring	Service satisfaction survey	1	Service performance	User satisfaction survey	1	Satisfaction	1
	Service linkage and termination	Cooperation with related institutions in community Providing information on service termination Storage of service provision documents	1 1 1	-	Connection with community Notice of service contract termination Storage of service provision documents	1 1 1	Community Contract termination Document filing	1 1 1
Field Evaluation Team	Organization chief’s leadership	Sense of duty and quality improvement in institutional operation Faithful preparation and creation of evaluation materials Consistency of self-evaluation report and evaluation materials	6	Overall evaluation	Organization chief’s efforts to improve service quality Degree of evaluation preparation Level of evaluation materials	2 2 2	Field evaluation	6
